# Generalized anxiety modulates frontal and limbic activation in major depression

**DOI:** 10.1186/1744-9081-8-8

**Published:** 2012-02-09

**Authors:** Michael W Schlund, Guillermo Verduzco, Michael F Cataldo, Rudolf Hoehn-Saric

**Affiliations:** 1 Department of Psychiatry, Johns Hopkins School of Medicine, 600 N. Wolfe St., Meyer 4-109, Baltimore MD 21287; 2 Kennedy Krieger Institute, 707 N. Broadway, Baltimore MD 21205; 3 Department of Behavior Analysis, University of North Texas, PO Box 310919, Denton TX 76203

## Abstract

**Background:**

Anxiety is relatively common in depression and capable of modifying the severity and course of depression. Yet our understanding of how anxiety modulates frontal and limbic activation in depression is limited.

**Methods:**

We used functional magnetic resonance imaging and two emotional information processing tasks to examine frontal and limbic activation in ten patients with major depression and comorbid with preceding generalized anxiety (MDD/GAD) and ten non-depressed controls.

**Results:**

Consistent with prior studies on depression, MDD/GAD patients showed hypoactivation in medial and middle frontal regions, as well as in the anterior cingulate, cingulate and insula. However, heightened anxiety in MDD/GAD patients was associated with increased activation in middle frontal regions and the insula and the effects varied with the type of emotional information presented.

**Conclusions:**

Our findings highlight frontal and limbic hypoactivation in patients with depression and comorbid anxiety and indicate that anxiety level may modulate frontal and limbic activation depending upon the emotional context. One implication of this finding is that divergent findings reported in the imaging literature on depression could reflect modulation of activation by anxiety level in response to different types of emotional information.

## Background

Depression is characterized by reductions in brain activation in the dorsal lateral and medial frontal cortex, the anterior cingulate and limbic structures [1-4]. Recent reports suggest that up to 12% of patients with major depressive disorder (MDD) are also comorbid with generalized anxiety disorder (GAD) [5]. Some hypothesize the early onset of GAD may confer vulnerability to later-developing disorders, including depression [6]. Comorbid anxiety may also modify the psychopathology and course of depression, with patients with agitated depression showing exacerbated bodily responses while patients with non-anxious depression manifest inhibitory autonomic responses [7]. Evidence also shows anxious arousal and anxious apprehension modulate activation differently in depression [1]. However, few neuroimaging investigations have examined brain activation in patients with depression and comorbid with preceding generalized anxiety (MDD/GAD). Such investigations are needed to ascertain whether frontal-limbic dysfunction common in depression is present and advance our understanding of the brain regions impacted by anxiety level.

Accordingly, we employed functional magnetic resonance imaging (fMRI) to examine the hypothesis that frontal-limbic dysfunction is present in a MDD/GAD group and anxiety level modulates regional brain activation, highlighting its importance as an individual difference variable. Frontal and limbic regions often associated with dysfunctional processing of emotional stimuli in depression were targeted. During imaging, subjects completed two affective challenges: one task that involved viewing facial expressions exhibiting anger, happiness or sadness and a second task involved hearing autobiographic sentences describing situations of ongoing significance that elicit anger, happiness or sadness. Brain activation was contrasted between the MDD/GAD group and a non-depressed control group and regions exhibiting hypoactivation in the MDD/GAD group were correlated with anxiety level. Importantly, this investigation specifically chose patients with major depression in whom chronic GAD preceded the first episode of depression in order to clarify the effects of anxiety on regional activation.

## Methods

### Participants

Ten unmedicated patients with clinical diagnoses of MDD comorbid with preceding GAD (MDD/GAD group: 4 males; mean age = 46.7 yrs, SD = 7.2 yrs) and 10 healthy controls (5 males; mean age = 40.0 yrs, SD = 10.2 yrs) recruited with advertisements gave oral and written consent to participate. All participants were right-handed and free of medications affecting the central nervous system for at least two weeks. MDD/GAD patients underwent a psychiatric and a physical examination that included the SCID-IV by a board-certified psychiatrist and a routine laboratory test with a urine toxicology screen to test for illicit drug use. Table 1 provides information on group characteristics. GAD preceded the first depressive episode with a mean onset of 24 years and the first episode of depression the age of 32.7 years. Onset of the present episode of depression was at 42.1 years. All subjects completed the Hamilton Depression Scale (HAM-D), Hamilton Anxiety Scale (HAM-A), and the State-Trait Anxiety Inventory (STAI) Trait form. Two-tailed Student t-tests confirmed that the MDD/GAD group showed significantly greater anxiety and depression ratings relative to the control group (*p's *< 0.005). Subjects were compensated $20 per hour. This study was approved by the Johns Hopkins Institutional Review Board for the Protection of Human Subjects.

**Table 1:** Group characteristics

		Years	Age of	Age of				
	Current	of	GAD	Depression	Age of Current			
**Group**	**Age (SD)**	**Education (SD)**	**Onset (SD)**	**Onset (SD)**	**Depression Onset (SD)**	**HAM-D**	**HAM-A**	**STAI**
Depressed	46.7 (7.2)	16.2 (2.1)	24 (14.1)	32.7 (15.4)	42.1 (11.3)	25.5 (6.2)	25.4 (5.6)	56.7 (5.3)
Controls	40.0 (10.2)	15.1 (3.2)	----	----	----	.2 (.3)	.7 (1.0)	29.7 (4.5)

### Neuroimaging Tasks

Two neuroimaging tasks were presented in random order, each during separate imaging runs lasting approximately 14 minutes. Task instructions were given verbally and printed on the subject's computer screen

#### Facial expressions

The task required subjects to view and categorize six *standardized *(3 male, 3 female) faces expressing anger, happiness or sadness [8]. Prior to imaging, two-sample t-tests on ratings of the intensity of emotional expressions revealed no group differences (*p's *> .05). Each trial lasted approximately 6.5 s and consisted of a 2 s period during which a single face was presented and a categorical response occurred, followed by 3-5 s blank screen. During presentation, subjects pressed one of three buttons, each corresponding to one type of expression. Each type of facial expression was presented for 40 trials within an event related design. Response accuracy for all subjects exceeded 80% correct and a two-sample t-test revealed no between-group difference (*p *> .05).

#### Autobiographic statements

The task required subjects to listen and categorize auditory descriptions of *personally relevant *situations. Prior to imaging, subjects were asked to provide six autobiographic statements describing two events that they believed would elicit anger, happiness and sadness. With assistance from subjects, statements were edited to fit within a 2 s time frame for the task. Each trial lasted approximately 6.5 s and consisted of a 2 s period during which one of the six statements was heard through headphones and a categorical response was made, followed by 3-5 s of silence. During presentation, subjects pressed one of three buttons, each corresponding to one type of emotion. Each type of statement was presented for 40 trials within an event related design. Response accuracy for all subjects exceeded 80% correct and a two-sample t-test revealed no between-group difference (*p *> .05).

### Functional neuroimaging acquisition and analysis

Functional MRIs were performed on a 3.0 T Phillips MRI, using a SENSE 8-channel head coil. Data were gathered using a single shot echo planar imaging (EPI) sequence for data acquisition, with a TR of 3 seconds, a TE of 35 ms and a 90 degree flip angle. The matrix size was 64 × 64 and the field of view 24 cm, yielding 3 mm isotropic voxels. Using these parameters, 20 whole brain contiguous axial sections were obtained angled parallel to the inter-commissural line. A high-resolution anatomical image was also acquired for co-registration with the functional scans. Images were acquired using a high resolution T1-weighted, 3D MP-RAGE sequence with a matrix size of 256 × 256, 150 axial slices, and 1 mm isotropic voxels. Preprocessing and group comparisons were performed using SPM software. Preprocessing included reorientation, slice acquisition time correction, co-registration with the structural image, realignment, spatial normalization to the standard Montreal Neurological Institute EPI template resampled to a 2 × 2 × 2 mm voxel size and spatial smoothing using a Gaussian kernel (6 mm fullwidth at half-maximum). High pass filtering was applied to remove any low frequency drift. A canonical hemodynamic response function was used as a covariate in a general linear model and a parameter estimate, which equates to percent change in the global mean signal, was generated for each voxel for each event type. At the first-level analysis, individual subject beta images were generated that were category-specific and consisted of the onsets of faces expressing anger, happiness and sadness and onsets of auditory descriptions of anger, happiness and sadness. Only trials with correct responses were used in the analyses. As our hypotheses focused solely on between group differences in frontal and limbic responses, category-specific images from each task generated at the first level were carried to a second-level analysis to perform between group comparisons using two-sample t-tests and the thresholds p < 0.001 uncorrected for multiple comparisons and 10 contiguous voxels. Afterwards, correlational analyses were performed using *p *< .05 to examine the relation between Hamilton Anxiety scores and activation restricted to frontal and limbic regions that evidenced significant between group differences.

## Results

### Group differences

**Figure 1:**
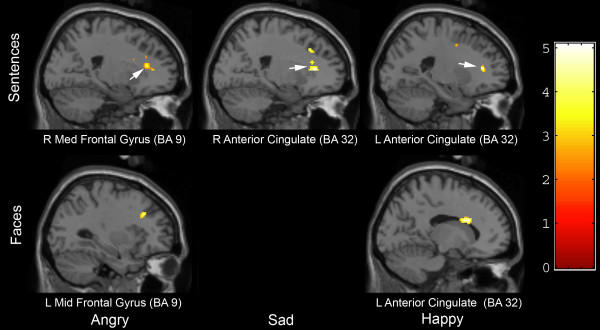
**Regions of hypoactivation exhibited by a group of depressed patients with comorbid with preceding generalized anxiety disorder compared to a group of non-depressed controls (*p *< .001)**. Group statistical parametric maps are arranged by stimulus presentation (rows: auditory autobiographical sentences, facial expressions) and type of emotional information (columns: angry, sad, happy).

Table 2 provides a complete listing of regions showing hyperactivation and hypoactivation in the MDD/GAD group relative to controls. Figure 1 highlights hypoactivation in frontal and anterior cingulate regions. Table 2 and Figure 1 highlight that statements were consistently associated with hypoactivation in the anterior cingulate across conditions and middle and medial frontal (BA 9) regions for angry and happy and the medial frontal region for sad. For facial emotions, hypoactivation in middle frontal regions (BA 9) was consistently observed across expressions, with additional hypoactivation observed in the insula and cingulate to negative facial expressions.

**Table 2:** Regional changes in activation in the MDD/GADgroup relative to a non-depressed control group.

Task	Increased activation				Decreased activation			
**Sentences**	**Region**	**Voxels**	**Talairach**	**Z**	**Region**	**Voxels**	**Talairach**	**Z**
Angry	L Culmen	40	-16 -51 -18	3.44	L Caudate	133	-12 2 11	4.3
	L Parahippocampus	63	-26 -32 -15	3.33	R Medial Frontal	58	24 32 15	3.53
	L Insula	52	-34 -22 20	3.29	R Anterior Cingulate	-	22 39 7	2.98
	L Superior Temporal	-	-38 -29 12	2.52	L Medial Frontal	57	-22 25 30	3.35
	L Claustrum	25	-22 23 -1	3.13	LCculmen	39	-2 -41 -3	3.13
	L Inferior Parietal	29	-46 -32 -24	2.87	L Thalamus	-	-2 -29 7	2.91
					R Middle Frontal	31	24 27 30	2.93
					L Cingulate	27	-4 23 39	2.67
Happy	L Amygdala	29	-32 -4 -12	3.34	L Anterior Cingulate	32	-22 35 9	4.15
	L Lingual gyrus	26	-12 -70 3	3.24	R Middle Frontal **	163	24 27 32	3.87
	R Precuneus	59	20 -67 24	3.2	R Anterior Cingulate	-	24 30 19	2.99
	R Posterior Cingulate	-	26 -61 20	2.94	L Cingulate	48	-16 6 46	3.72
	R Middle Temporal	-	30 -57 23	2.43	L Middle Frontal **	-	-24 2 40	2.93
	R Thalamus	35	20 -20 -2	2.83	L Caudate	31	-10 1 13	3.49
	R Putamen	-	26 -21 1	2.6	L Inferior Frontal	42	-40 3 27	3.31
	R Culmen	25	6 -35 -2	2.8				
Sad	R Superior Temporal	51	42 -27 3	3.53	R Anterior Cingulate	80	22 32 9	3.38
					R Claustrum	-	24 26 8	3.18
					R Medial Frontal	32	22 27 32	2.99
Faces								
Angry					L Inferior Parietal	84	-38 -31 31	3.95
					R Insula	28	34 -25 9	3.74
					L Middle Frontal **	41	-30 29 28	3.62
					L Insula**	-	-34 26 17	2.95
					R Cingulate	54	18 -6 43	3.21
Happy	L Posterior Cingulate	82	-10 -44 13	3.71	R Middle Frontal	11	32 13 27	5.57
	L Parahippocampus	-	-14 46 6	2.82				
	L Inferior Parietal	36	-28 -51 32	3.55				
	R Lingual gyrus	69	4 -80 1	3.34				
	R Cuneus	-	12 -83 10	2.51				
	R Posterior Cingulate	33	6 -56 5	3.22				
	R Lingual gyrus	46	14 -70 2	3.1				
	R Cingulate	52	8 -27 36	3.06				
Sad					L Inferior Parietal	83	-40 -31 31	3.95
					R Inferior Parietal	28	34 -31 38	3.74
					R Insula **	41	-30 25 9	3.62
					L Middle Frontal	-	-34 29 28	2.95
					L Insula	54	16 26 17	3.21
					R Cingulate	-	18 -6 43	2.99

** Regions showing a positive correlation with anxiety.

### Brain-behavior relationships

Highlighted in Table 2 are regions that were positively correlated with anxiety level. For statements, anxiety levels were positively correlated with activation to happy sentences in the right (r = .56) and left middle frontal gyrus (r = .56). Anxiety levels were also positively correlated with activation to angry faces in the left middle frontal gyrus (r = .48) and the left insula (r = .55).

## Discussion

Two notable findings emerged from this preliminary investigation that contribute to clinical neuroscience research on depression. First, the relatively consistent pattern of hypoactivation seen in the MDD/GAD group in medial and middle frontal regions as well as in the anterior cingulate, cingulate and insula is generally consistent with prior findings demonstrating frontal-limbic dysfunction in depression [2-4]. Reduced activation in MDD/GAD patients was found in the insula and right cingulate gyrus for both negative visual stimuli but not the positive stimulus, suggesting a decrease in somato-affective [9] and cognitive-attentional interactions [10]. One interpretation of the hypoactivation observed in medial/middle frontal regions is that it reflects reduced engagement with provocative, emotional stimuli. The middle frontal gyrus has been associated with sustained attention, working memory and language [11], thus the reduced activation suggests a decrease in executive functioning [12]. Presentations of individualized autobiographic statements were also associated with attenuated responses in the dorsal anterior cingulate cortex, an area associated with cognitive-emotional responses [13]. These results demonstrate comorbid anxiety may not work in opposition to depressive symptoms in ways that minimize between group differences commonly reported. It should be noted that we did not observe activation in the ventromedial and subgenual cingulate cortex or amygdala [2,4] during presentation of standardized visual stimuli. Whether this difference reflects a procedural difference or the effects of a prolonged period of GAD is unclear.

The second notable finding of this investigation concerns modulation of activation by anxiety level during emotional information processing. In regions that showed hypoactivation, increased anxiety was correlated with increased activation in middle frontal regions and the insula but only during presentation of faces expressing anger and autobiographical sentences describing happy personal events, suggesting that anxiety effects do not generalize across all types of emotional information. The area of insula activation observed was more posterior in our study compared with other anxiety studies [14]. The positive correlation between insula activation and anxiety level suggests that the posterior part of the insula also may be involved in the neurobiology of anxiety. One implication of these findings for research on depression is that anxiety level may serve as an important individual difference variable [1]. Complicating matters is that anxiety level does not equally modulate activation to all types of emotional information. The important implication is that divergent findings reported in the imaging literature on depression could reflect modulation of activation by anxiety level in response to different emotional information.

While our findings suggest frontal and limbic dysfunction in patients with depression and comorbid anxiety and modulation of activation by anxiety level, additional research including larger samples of patients with major depression alone and with comorbid generalized anxiety is needed to replicate findings and clarify the effects of anxiety. Future research is needed that refines the assessment of manifestations of anxiety in novel ways that extend beyond DSM categorizations, such as considering measures of negative forecasting, avoidance frequency, level of experiential avoidance and sustained levels of activation, The integration of alternative measures of anxiety with neuroimaging technology may provide additional novel insights into the brain abnormalities underlying depression.

## Conclusion

Our findings highlight frontal and limbic hypoactivation in patients with depression and comorbid with preceding anxiety and indicate that anxiety level may modulate frontal and limbic activation depending upon the emotional context. One implication of this finding is that divergent findings reported in the imaging literature on depression could reflect modulation of activation by anxiety level in response to different emotional information.

## Competing interests

The authors declare that they have no competing interests.

## Authors' contributions

Design: R.H-S Data collection: M.S., R.H-S. Analysis: G.V., R.H. Writing: M.S., R.H-S. M.C. All authors read and approved the final manuscript.
